# The Structure of Mindful Brain

**DOI:** 10.1371/journal.pone.0046377

**Published:** 2012-09-28

**Authors:** Hiroki Murakami, Takashi Nakao, Masahiro Matsunaga, Yukinori Kasuya, Jun Shinoda, Jitsuhiro Yamada, Hideki Ohira

**Affiliations:** 1 Department of Clinical Neuroimaging, Integrative Brain Imaging Center, National Center of Neurology and Psychiatry, Tokyo, Japan; 2 Institute of Biomedical and Health Sciences, Hiroshima University, Hiroshima, Japan; 3 Division of Cerebral Integration, Department of Cerebral Research, National Institute for Physiological Sciences, Okazaki, Japan; 4 Kizawa Memorial Hospital, Chubu Medical Center for Prolonged Traumatic Brain Dysfunction, Minokamo, Japan; 5 Department of Psychology, Graduate School of Environmental Studies, Nagoya University, Nagoya, Japan; University of Medicine & Dentistry of NJ - New Jersey Medical School, United States of America

## Abstract

Mindfulness is currently attracting a great deal of attention as a psychotherapy technique. It is defined as bringing one's complete attention to the experiences occurring in the present moment in a nonjudgmental or accepting way. The Five Facet Mindfulness Questionnaire (FFMQ) was developed to assess individual differences in mindfulness states. The FFMQ is composed of five facets representing elements of mindfulness: non-reactivity to inner experience, non-judging, acting with awareness, describing, and observing. In the present study, we applied voxel-based morphometry to investigate the relationship between the brain structure and each facet as measured by the FFMQ. The results showed a positive association between the describing facet of mindfulness on the FFMQ and gray matter volume in the right anterior insula and the right amygdala. In conclusion, mindfulness was related with development in parts of the somatic marker circuit of the brain.

## Introduction

Mindfulness is currently attracting a great deal of attention as a psychotherapy technique. The technique originated from Buddhist meditation practices, and is defined as bringing one's complete attention to the experiences occurring in the present moment in a nonjudgmental or accepting way [1]. Mindfulness practice is hypothesized to develop a distanced or decentered relationship with one's internal and external experiences, which decreases emotional reactivity [2]. In a one-year follow-up study, 8 week mindfulness-based cognitive therapy had efficacy in reducing the relapse/recurrence of major depression compared with the usual treatment, in which patients were instructed to seek help from their family doctor, or other sources, as they normally would [3]. Another one-year follow-up study with breast and prostate cancer patients showed biological efficacy, in that mindfulness intervention reduced the levels of inflammatory cytokines and cortisol, which is a stress hormone, as well as stress symptoms [4]. Mindfulness is effective not only as a practice and therapeutic intervention, but is considered to have a tendency to stable individual differences. To assess mindfulness states, Baer et al. [1] developed a 39-item instrument, named the Five Facet Mindfulness Questionnaire (FFMQ), which is composed of five facets: non-reactivity to inner experience, non-judging of inner experience, acting with awareness, describing, and observing.

Few studies have investigated the relationship between mindfulness meditation and brain structures. Lazar et al. [5] showed that meditators whose practice included mindfulness factors showed thicker gray matter in the right anterior insula than a control group. The right anterior insula contains interoceptive re-representations that substantialize all subjective feelings from the body and perhaps emotional awareness [6]. Additionally, in a longitudinal study, a reduction in perceived stress by mindfulness-based stress reduction intervention for 8 weeks was correlated with a change in gray matter density within the right amygdala [7]. The amygdala is well known to play a primary role in psychological and physiological emotional responses [8]. Furthermore, Luder, Toga, Lepore and Gaser [9] showed that meditators have greater gray matter volume in the right orbitofrontal cortex, and Hölzel et al. [10] demonstrated that the gray matter concentration in meditators was greater in the right anterior insula and right hippocampus. The orbitofrontal cortex is related with emotion and emotion regulation [11], and gray matter volume in the hippocampus declines by chronic stress [12]. Hölzel et al. [13] examined differences in brain structure between participants received an 8-week mindfulness training program and a waiting list control group. Whole brain analyses identified increases in the posterior cingulate cortex, the temporo-parietal junction, and the cerebellum in the mindfulness training group compared with the controls. The mindfulness training program is associated with changes in gray matter concentration in brain regions involved in learning and memory processes, emotion regulation, self-referential processing, and perspective taking. These previous studies showed that mindfulness meditation is related with brain structures in distributed regions of the brain: the right anterior insula, amygdala, orbitofrontal cortex, hippocampus, posterior cingulate cortex, temporo-parietal junction, and cerebellum.

As described above, mindful is considered to consist of five facets. It is known that the gray matter volume is developed by learning in the domains of the learning-related brain regions [14], [15], [16]. Thus, it can be considered that each facet of mindfulness might be related with development of the gray matter volume in different brain regions, as suggested by the previous findings. Therefore, in the present study, we investigated the relationship between brain structures and each facet of mindfulness as measured by the FFMQ.

## Methods

### Participants

The participants were 19 undergraduate and graduate student volunteers (12 men and 7 women; age range, 18–24 years; mean age, 21.2 years, SD = 1.9) recruited from Nagoya University. All participants were Japanese, right-handed and had normal vision. They were free of neurological or psychiatric disorders. All the participants provided written informed consent in accordance with the Declaration of Helsinki. This study was approved by the Ethics Committee of Kizawa Memorial Hospital.

### Assessment of Mindfulness

The Japanese version of the FFMQ [17], which was originally developed by Baer et al. [1], was used to assess the five facets in each individual. The resulting instrument is a 39-item, Likert-type measure assessing five identified facets of mindfulness: nonreactivity: nonreactivity to inner experience; observing: noticing and paying attention to one's own emotions, feelings, body experience, and behavior; acting with awareness/automatic pilot/concentration/nondistraction; describing: finding the words to describe one's own feelings; nonjudging of experience.

### MRI data acquisition and analysis

A high-resolution T1-weighted whole-brain image was collected for every subject on a 1.5-T MRI scanner (SIGNA MR/I Echo Speed 1.5 T CV, NV Option; General Electric Medical Systems) with the following parameters: echo time (TE) = 1.6 ms, repetition time (TR) = 7.2 ms, flip angle = 20°, matrix size = 256×256, field of view (FOV) = 240 mm, slice thickness = 1.0 mm, interslice gap = 0 mm. Within the TR at each time point, 164 slices of the brain were acquired axially. Head movements were restricted by stabilizing the head with tape and cushions; we tightened the tape to the extent that the participants did not feel distress.

Image analysis and processing were performed following the optimized voxel-based morphometry procedure [18] by using SPM5 software available at (http://www.fil.ion.ucl.ac.uk/spm) on the Matlab R2007a platform applying the VBM toolbox (version 5.1; http://dbm.neuro.uni-jena.de/vbm). T1- weighted images from the subjects were prepared for VBM analyses using a fully automated algorithm script in Matlab. Raw images from the scans of all subjects were normalized to the standard Montreal Neurological Institute (MNI) T1 template provided by SPM 5. Normalized images were then segmented into their gray matter, white matter, and cerebrospinal fluid components with an automated algorithm [19]. Finally, the whole brain template and all segmented tissue templates were smoothed with a 12- mm full-width at half-maximum (FWHM) isotropic Gaussian kernel.

Correlations between brain structures and each facet of the mindfulness tendency score were explored across the whole brain using voxel-based morphometry with age and sex entered as covariates. Because of our a priori hypothesis targeting the right anterior insula, amygdala, orbitofrontal cortex, hippocampus, posterior cingulate cortex, temporo-parietal junction, and cerebellum, a less conservative threshold (an inclusion threshold of p<0.005 uncorrected with an extent threshold of at least 125 contiguous voxels) was applied. The Mascoi toolbox for SPM 5 [20] was used to label the clusters.

## Results

**Figure 1:**
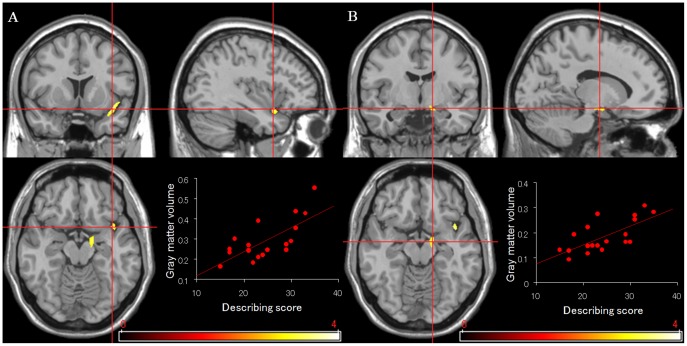
Correlations of the score of the describing facet in mindfulness tendency and gray matter volume. Results of correlation analyses showing the positive correlations between the score of the describing facet and the gray matter volume in the right anterior insular cortex (A), and in the right parahippocampal gyrus/amygdala (B). An uncorrected *p* value of 0.005 was used as the threshold.

A multiple regression analysis in SPM5 with covariate control for age and sex revealed that gray matter volume is significantly associated with a facet of the mindfulness tendency (see Table 1). The score of the describing facet is positively associated with the right anterior insular cortex, and right parahippocampal gyrus/amygdala (Fig. 1). No other facets of the mindfulness tendency were correlated with gray matter volume. There were no regions which were negatively correlated with any facets of the mindfulness tendency.

**Table 1:** Regions of gray matter positively correlated with each facet of mindfulness tendency.

Region	Brodmann areas	Side	Coordinates			Cluster size	*t* value
			*x*	*y*	*z*		
Correlated with describing facet							
Anterior insular cortex	13	Right	37	10	−15	442	3.72
Parahippocampal gyrus/amygdala	28	Right	13	−7	−14	192	3.53

## Discussion

In this study, we investigated the association of the mindfulness tendency as assessed by the Japanese version of the FFMQ and regional gray matter volume in the human brain. We found a positive association between the describing facet in mindfulness tendency and gray matter volume in the right anterior insula and right amygdala. These findings were consistent with our hypothesis. The greater volume of the right anterior insula shown in individuals with a higher describing score in the present study is compatible with the previous finding in extensive meditation practitioners [5]. The right anterior insula contains interoceptive re-representations that substantialize all subjective feelings from the body and emotional awareness [6]. This function of this region is consistent with the definition of the describing facet of the mindfulness tendency, and is contrary to the description of alexithymia, a reduction or incapacity to experience or verbalize emotions [21]. The developed right anterior insula volume in individuals with a higher describing score in the present study may facilitate more awareness of their own emotional states, and awareness of their own stressful states may enable them to exert cognitive control over their emotions.

Additionally, a previous study demonstrated that the amygdala volume is negatively associated with amygdala activity and blood pressure during a stress task [22]. This suggested that the greater amygdala volume in individuals with higher describing scores would result in lower activity in the amygdala and subsequent lower blood pressure. The amygdala is under tonic inhibitory control via γ-aminobutyric acid-ergic mediated projections from the prefrontal cortex [23], [24]. Animal studies have suggested that the basolateral amygdala receives inhibitory input from the prefrontal cortex [25]. Taken together, the developed amygdala volume, if it includes the basolateral amygdala, in individuals with higher describing scores in the present study may allow superior cognitive control of emotional responses by the prefrontal cortex. The amygdala and anterior insula related with the describing facet of mindfulness tendency are parts of the somatic marker circuit. The amygdala is trigger of emotional/bodily states in response to environment, and the anterior insula is mapping of bodily/emotional states gives rise to conscious “gut feelings” [26].

The present study has several limitations. First, we could only find an association between the gray matter volume and mindfulness tendency in a facet. The other facets of the mindfulness tendency may not be related with the gray matter volume. Second, we could not find any association between the mindfulness tendency and orbitofrontal cortex, hippocampus, posterior cingulate cortex, temporo-parietal junction, and cerebellum. We investigated a young population, but the gray matter volume declines by aging [27]. Therefore, it may be possible to see the effects of other facets of the mindfulness tendency on brain structures and the association between the mindfulness tendency and orbitofrontal cortex, hippocampus, posterior cingulate cortex, temporo-parietal junction, and cerebellum in an elderly population. The present study is a cross-sectional one, and we therefore had limited ability to predict a causal relationship between the mindfulness tendency and brain structures. To solve this limitation, further research is needed using longitudinal studies to investigate brain structure alteration between pre-mindfulness training and post mindfulness training. Additionally, it is necessary to investigate the association between the mindfulness tendency and brain activation and behavioral performance while conducting cognitive tasks in addition to gray matter volume.

In conclusion, we could demonstrate our hypothesis that mindfulness is related with brain structures in the right anterior insula and amygdala. To our knowledge, this is the first study to show the relationship between the mindfulness tendency measured by the FFMQ and gray matter volume.
